# A Digital PCR-Based Method for Efficient and Highly Specific Screening of Genome Edited Cells

**DOI:** 10.1371/journal.pone.0153901

**Published:** 2016-04-18

**Authors:** Scott D. Findlay, Krista M. Vincent, Jennifer R. Berman, Lynne-Marie Postovit

**Affiliations:** 1 Department of Anatomy and Cell Biology, Faculty of Medicine and Dentistry, University of Western Ontario, London, Ontario, Canada; 2 Department of Oncology, Faculty of Medicine and Dentistry, University of Alberta, Edmonton, Alberta, Canada; 3 Digital Biology Center, Bio-Rad Laboratories, Pleasanton, California, United States of America; SRI International, UNITED STATES

## Abstract

The rapid adoption of gene editing tools such as CRISPRs and TALENs for research and eventually therapeutics necessitates assays that can rapidly detect and quantitate the desired alterations. Currently, the most commonly used assay employs “mismatch nucleases” T7E1 or “Surveyor” that recognize and cleave heteroduplexed DNA amplicons containing mismatched base-pairs. However, this assay is prone to false positives due to cancer-associated mutations and/or SNPs and requires large amounts of starting material. Here we describe a powerful alternative wherein droplet digital PCR (ddPCR) can be used to decipher homozygous from heterozygous mutations with superior levels of both precision and sensitivity. We use this assay to detect knockout inducing alterations to stem cell associated proteins, NODAL and SFRP1, generated using either TALENs or an “all-in-one” CRISPR/Cas plasmid that we have modified for one-step cloning and blue/white screening of transformants. Moreover, we highlight how ddPCR can be used to assess the efficiency of varying TALEN-based strategies. Collectively, this work highlights how ddPCR-based screening can be paired with CRISPR and TALEN technologies to enable sensitive, specific, and streamlined approaches to gene editing and validation.

## Introduction

### Utility of genome editing

Genome editing is already transforming how molecular biological research is conducted. The ability to precisely modify the genome in a targeted fashion has numerous applications in molecular biology. These include the functional knockout of endogenous genes or alleles, and the targeted induction or correction of specific mutations or other polymorphisms. In addition to allowing precise modelling of genetic contributions to normal cellular function, genome editing has obvious potential as a therapeutic tool for the treatment of a wide range of diseases.

### Types of technologies

The advent of the transcription activator-like effector nuclease (TALEN) and more recently, clustered regularly interspaced short palindromic repeats (CRISPR-Cas) systems have accelerated the adoption of genome editing approaches in many fields of molecular biology [1–4]. TALEN nucleases consist of a modular DNA binding domain fused to an endonuclease domain to induce double stranded breaks at a DNA target. CRISPR-Cas systems rely on base pairing between an exogenous guide RNA (gRNA) and an endogenous DNA target to deliver the CRISPR-associated (Cas) endonuclease. Regardless of the genome editing system used, the generation of a double stranded break is repaired by the cell using either the non-homologous end joining (NHEJ) pathway, or homology-directed repair (HDR). NHEJ is error-prone and frequently generates small indel mutations [5]. Such mutations are easily exploited for functional gene knock-out studies in human cell lines as well as various model organisms [6–8].

### Demand for rapid cloning

The widespread use of TALEN and CRISPR gene editing systems has been accelerated by the generation and availability of a wide variety of rapid cloning systems and expression plasmids. The availability of diverse expression systems is important to meet the demands of different projects and users. For the CRISPR-Cas system, the simplest system is a single “all-in-one” plasmid coding for both the Cas protein and a gRNA that is easily modified, as well as a mammalian antibiotic resistance gene and/ or fluorescent protein for enrichment of edited cells. Here we describe an “all-in-one” CRISPR/Cas plasmid that we have modified for one-step cloning and blue/ white screening of transformants. We have utilized this plasmid as part of the rapid and quantitative genome editing workflow we describe here.

### Demand for quantitative screening

For any genome editing application, the reliable detection of nuclease-induced mutations is paramount to moving forward with a project. Furthermore, there is a great demand for mutation detection assays to be quantitative, sensitive, and universal in that they can be readily adapted to any target of interest. Genome editing experiments often result in low mutation frequencies in bulk populations of treated cells. Therefore precise quantification of mutation rates is extremely important for optimization of genome editing protocols and downstream workflow, such as determining how many single cell-derived clones to screen for desired mutations.

### Common screening assays

While next generation sequencing offers a gold standard for quantitative determination of nuclease-induced mutation detection, such approaches are often not practical. Many different methods to screen for nuclease-induced mutations have been reported [9–12]. However, the most widely used assays to screen for mutations utilize the so-called “mismatch nucleases” T7E1 or “Surveyor” that recognize and cleave heteroduplexed DNA amplicons containing mismatched base-pairs [13]. However, these assays have several shortcomings. First, they require a relatively large amount of starting material to generate at least 200 ng (and ideally up to 500 ng) of purified PCR product corresponding to the target locus. This requirement does not allow for rapid workflows, as significant cellular expansion is needed after enriching for editing cells using selection or sorting, and again after the generation of single cell-derived clones. Second, there are obvious limitations for sensitivity, as digested fragments that do not make up a large portion of the total amplified target molecules are hard to distinguish from background noise on an electrophoretic gel. Furthermore, targets that cannot be efficiently amplified may not result in bright bands. Indeed, due to the nature of intercalating DNA stains, each digested fragment loses a minimum of 50% of its signal relative to its parent band. Third, this method has very limited utility for screening of single-cell derived clones. For a typical diploid target locus, a clone with both alleles successfully mutated by NHEJ, but containing distinct indels, will be indistinguishable from a clone with one mutated allele and one wild type allele, as each of these samples would contain a 50–50 mix of distinct alleles. In both cases, half of the duplexed DNA would be in the heteroduplexed form, with the remaining half forming homoduplexes (25% allele one only and 25% allele two only) that are not cleaved by the endonuclease. This inability to discriminate the highly desirable clones with two mutated indel alleles from those with only one mutated allele translates to a larger investment of resources by the user—more sequencing and/ or additional screening of candidate clones. Fourth, these assays generally require the generation of amplicons of at least 400 base pairs to ensure digested fragments are of sufficient length to be visualized. This increases the chances of the amplicon encompassing a polymorphism that is heterozygous in the sample or cell line being used. An endogenous heterozygous SNP or mutant allele anywhere in the amplicon can be recognized by the nuclease and lead to a false-positive signal, even in unedited cells. This is especially problematic in cancer cell lines and samples where mutation frequencies are extremely high, change dynamically, and are often unknown for a particular locus of interest.

### Droplet digital PCR

The emergence of droplet digital PCR (ddPCR) methods provide a new opportunity for mutation screening that provides superior sensitivity, is absolutely quantitative, and can easily be adapted to any target of interest. Quantification of NHEJ-induced indels as well as donor-derived mutations of interest using droplet digital PCR has just recently been reported [14,15]. Due to the ability to obtain absolute quantifications from very small amounts of DNA, this methodology holds great promise as a preferred method of screening. However, the utility and performance of such assays have not yet been thoroughly assessed.

In this paper, we provide the first thorough report on droplet digital PCR assays for the detection of NHEJ-induced mutations, using stem cell-associated genes known to be activated in various cancers as example targets. We detail the design, optimization, performance, and utility of these assays across multiple genomic targets using both CRISPR-Cas and TALEN gene editing systems. We demonstrate that droplet digital PCR assays are more accurate, sensitive, and adaptable to a given target than mismatch nuclease assays, and can serve as a platform for the quantitative comparison of different genome editing platforms, approaches, and protocols.

## Results

We chose the stem cell-associated genes nodal growth and differentiation factor (*NODAL*) and secreted frizzled-related protein 1 (*SFRP1*) known to play important roles in the pathology of numerous cancers [16,17] as example targets for testing the utility of droplet digital PCR-based mutation screening assays. We chose these targets as practical candidates for functional knock-out using precision nucleases to target the first coding exon of these genes. We employed a typical workflow for nuclease-based gene knock-out: Bulk transfected and selected populations were screened for induced mutations within the target region. Single-cell derived clones were then generated and screened for desired indel mutations at both alleles.

### An all-in-one CRISPR plasmid for one step gRNA cloning

**Fig 1:**
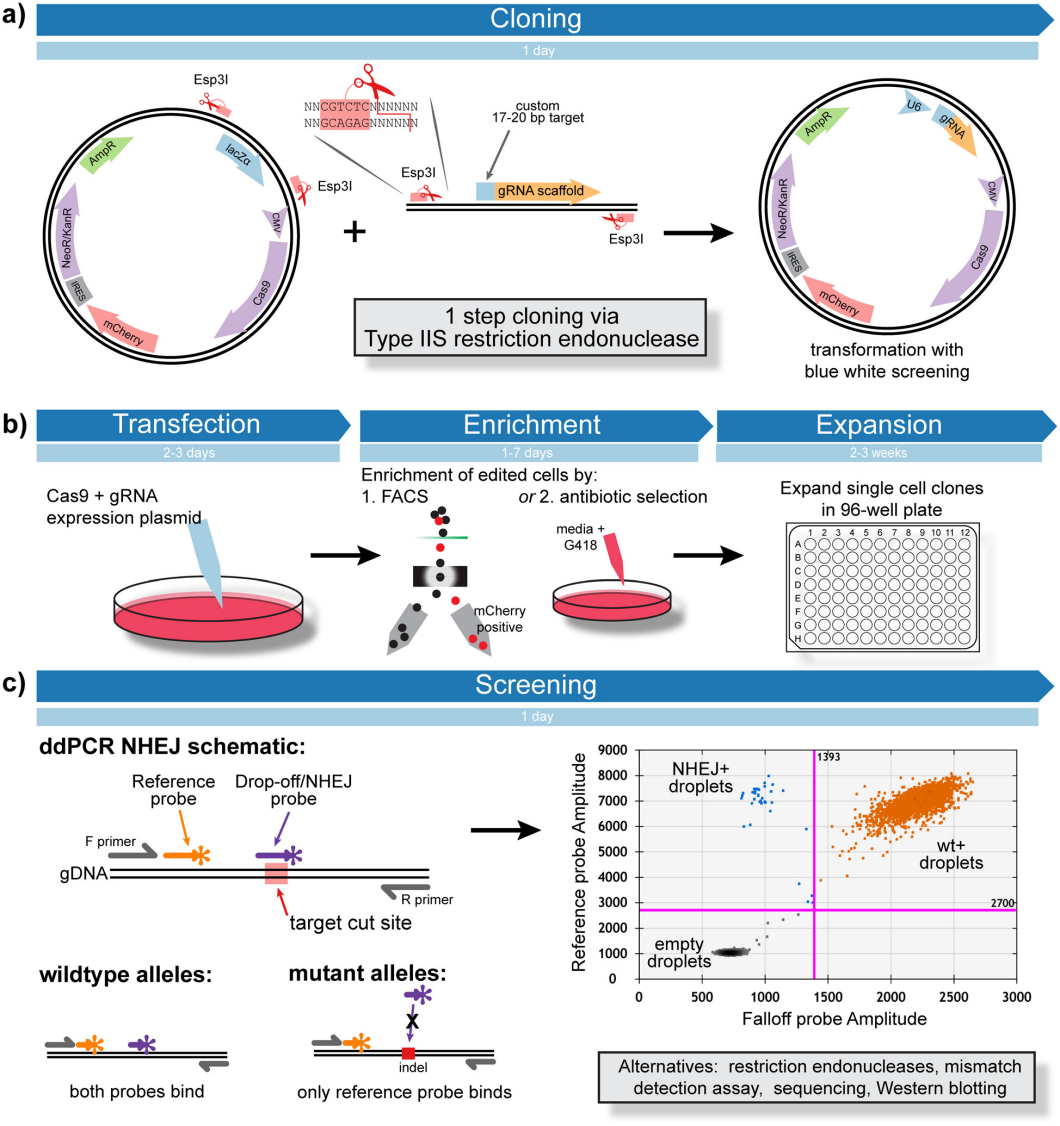
CRISPR workflow. (a) Use of type IIS restriction enzymatic digestion enables rapid cloning of a custom CRISPR gRNA in a plasmid containing Cas9, mCherry and Neo resistance cassettes. Transformants of recombinant plasmids will be white, while transformants of non-recombinant plasmids will be blue. (b) Target cell lines are transfected with the Cas9+gRNA expression plasmid, enriched by cell sorting or antibiotic selection and expanded as single cell clones. (c) Schematic of droplet digital PCR-based screening of successful NHEJ.

For CRISPR/Cas-based editing, we first adapted an all-in-one CRISPR/Cas plasmid for rapid cloning using the type IIS restriction enzyme Esp3I and traditional blue/ white screening by inserting a LacZ-α open reading frame between two newly generated Esp3I restriction sites (Fig 1A). This plasmid allows for rapid single step cloning of any desired gRNA. It is a single plasmid system containing a mammalian antibiotic resistance gene and mCherry fluorescent protein for enrichment of transfected cells, if desired (Fig 1B). For TALEN-based editing, we used cloning methods previously described (see methods).

### Mutation detection using droplet digital PCR assays

**Fig 2:**
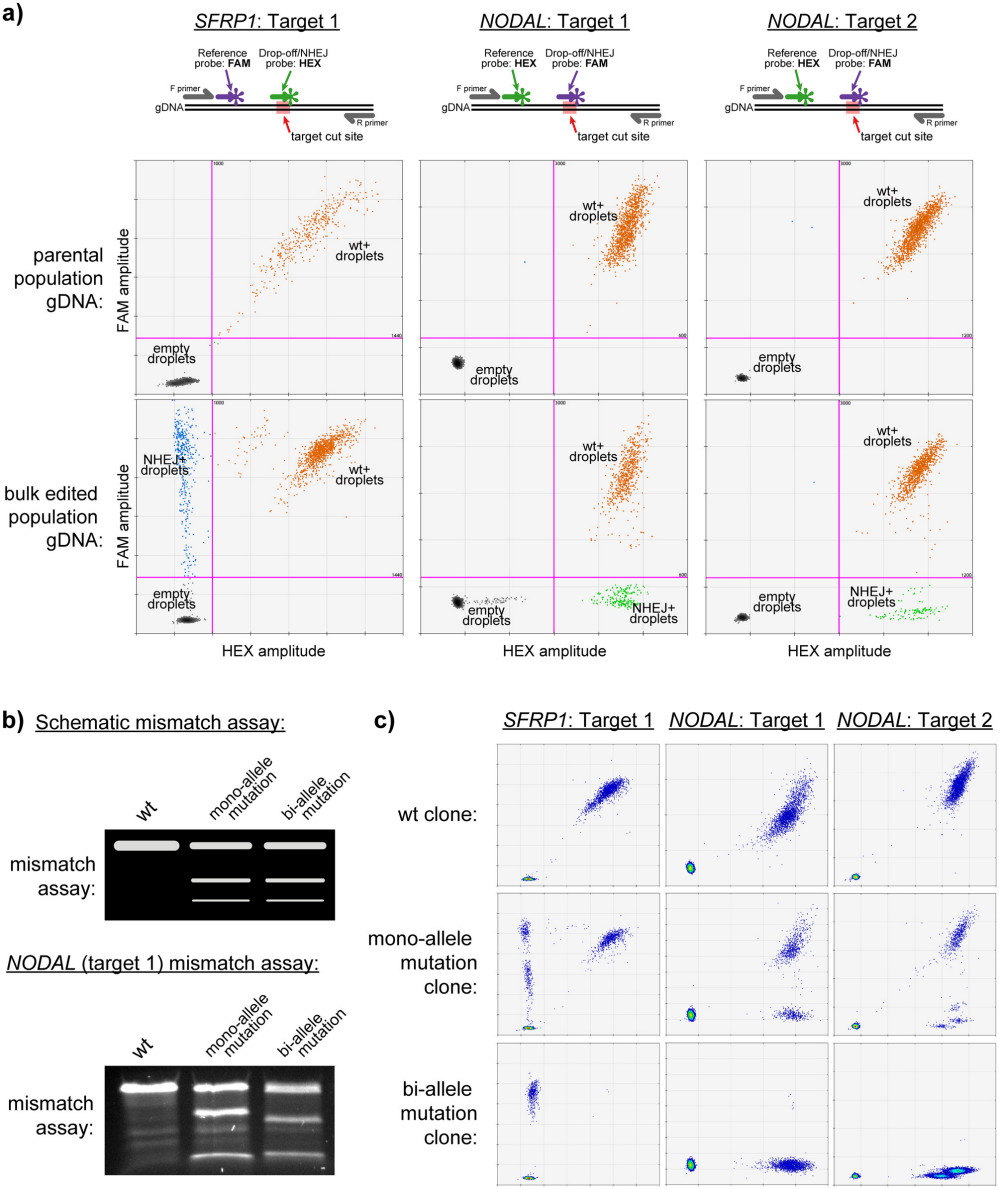
Droplet digital PCR can be used to screen for indels in bulk-edited populations and single cell clones. (a) Example ddPCR plots of parental population gDNA and bulk-edited population gDNA for three given genomic targets. (b) Conceptual and actual results of a T7E1 digestion resolved by gel electrophoresis for wildtype, mono-allelic mutant and bi-allelic mutant clones. (c) ddPCR plots of wildtype, mono-allelic mutant and bi-allelic mutant clones. Note that FAM and HEX are used for different probes between the *SFRP1* and *NODAL* assays.

We demonstrate the ability of ddPCR-based assays to successfully detect nuclease-induced mutations in our sample targets. These assays consist of a duplexed primer probe based ddPCR assay in which one probe binds a “reference” sequence distant from the nuclease target site but still within the amplicon, and a second “NHEJ/drop-off” probe binds at the nuclease target site. In a 2-dimensional view of the ddPCR analysis, droplets containing signal from both probes contain wild-type amplicons, while droplets containing signal from the reference probe but not the NHEJ/drop-off probe contain amplicons with mutations at the target site (Fig 1C). We very rarely detected droplets that could be classified as containing target mutations in control-transfected or parental samples, and successfully detected nuclease-induced mutations in CRISPR or TALEN-treated cells (Fig 2A). For single cell-derived clones, the same assays reliably detected induced mutations. Unlike mismatch nuclease assays, our ddPCR assays were definitively able to distinguish samples with mono-allelic versus bi-allelic mutations (Fig 2B and 2C). All ddPCR assays were validated by sequencing single cell-derived clones (see methods). No mutant sequences were detected in samples with virtually all droplets clustering as double positive/ wild type. Both mutant sequences and wild type sequences were detected in samples with both wild type and NHEJ droplets. No wild type sequences were detected in samples with virtually all droplets clustering as NHEJ droplets (Fig 2C).

### Performance of ddPCR mutation assays

**Fig 3:**
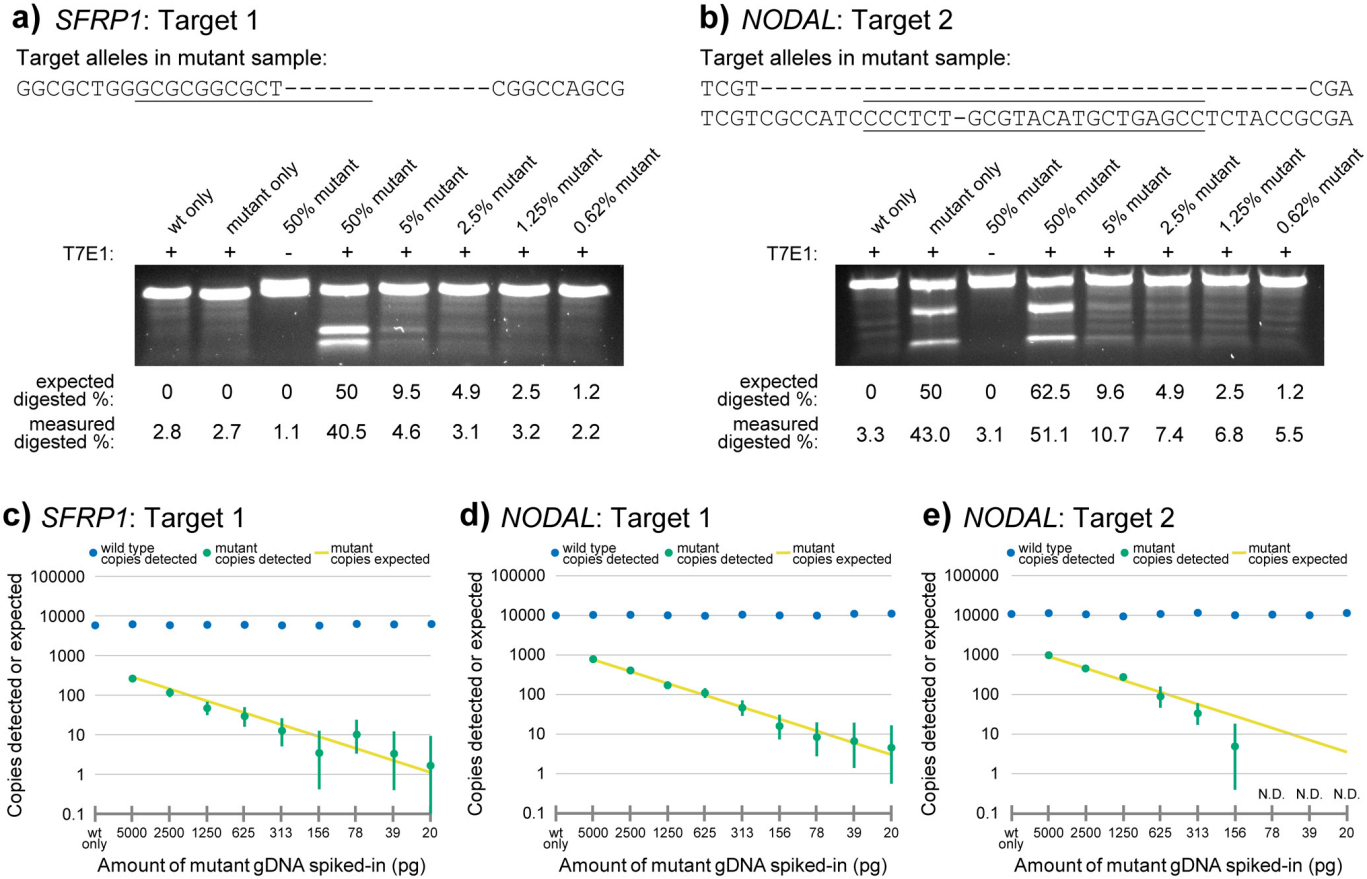
Sensitivity of indel detection in mismatch and ddPCR assays. (a,b) Genomic DNA samples from wild type and target-disrupted cells were mixed in indicated ratios and subjected to a T7E1 cleavage assay. Gel analysis was conducted on *SFRP1* (a) and *NODAL* (b) targets. DNA sequences of the mutant cellular clone are displayed at the top of the panel. Underlines indicate TALEN spacer region in a, and gRNA target (including PAM sequence) in b. (c-e) Genomic DNA from target-disrupted samples were added to wild type samples at indicated amounts and subjected to ddPCR analysis. Error bars indicate 95% confidence interval. Yellow line depicts the mutant copies expected. “N.D.” indicates none detected.

We tested the performance of these assays by spiking in genomic DNA (gDNA) from a single-cell derived clone with bi-allelic mutations into a high concentration of non-mutated wild type gDNA. This allowed us to create carefully controlled samples representing a small number of mutated cells in a larger background of non-mutated cells, while maintaining the natural complexity, concentration, and purity of a typical gDNA sample. These samples were then subjected to both our ddPCR assays and standard mismatch nuclease assays for performance evaluation (Fig 3).

### Sensitivity

The mismatch nuclease assays were not very sensitive, and concentrated purified PCR product is required as input. In our assays, 0.6% (2.5 ng mutant in 400 ng total PCR product) mutant DNA was difficult to distinguish from background noise for both targets (Fig 3A and 3B). Thus we did not test dilutions below 0.6%. The absolute sensitivity of these assays is very poor (0.6% is 2.5 ng of mutant PCR product, which is approximately 4 x 10^9^ copies of DNA), despite the large amount of input gDNA required to generate sufficient PCR product. In our ddPCR assays, we were able to successfully detect a minimum of between 20 pg and 156 pg of mutant genomic DNA (not purified PCR product) in a high background of 100 ng of wild type gDNA for our three targets (Fig 3C–3E). We did not test below 20 pg as this amount of gDNA was expected to contain between only 1 and 4 copies of target DNA (see methods) and thus served as a practical lower limit.

### Accuracy

Our mismatch nuclease assays were not very accurate in their quantification of mutant PCR product at any of the dilutions tested (Fig 3A and 3B). In the corresponding ddPCR assays, the 95% confidence intervals for the detected copies of mutant gDNA generally encompassed the expected number of mutant copies from 5 ng to 20 pg of mutant genomic DNA (Fig 3C–3E). Furthermore, the amount of wild type gDNA detected by the ddPCR assays remained stable across samples and was not affected by the amount of mutant gDNA loaded as there was no significant correlation between mutant gDNA loaded and copies of wild type detected (correlation coefficient r^2^ = 0.002 for *SFRP1* target 1, r^2^ = 0.014 for *NODAL* target 1, and r^2^ = 0.045 for *NODAL* target 2). These data demonstrate that these assays are capable of accurate quantitative detection of extremely rare mutations in 100 ng of gDNA.

### Utility of ddPCR mutation assays

**Fig 4:**
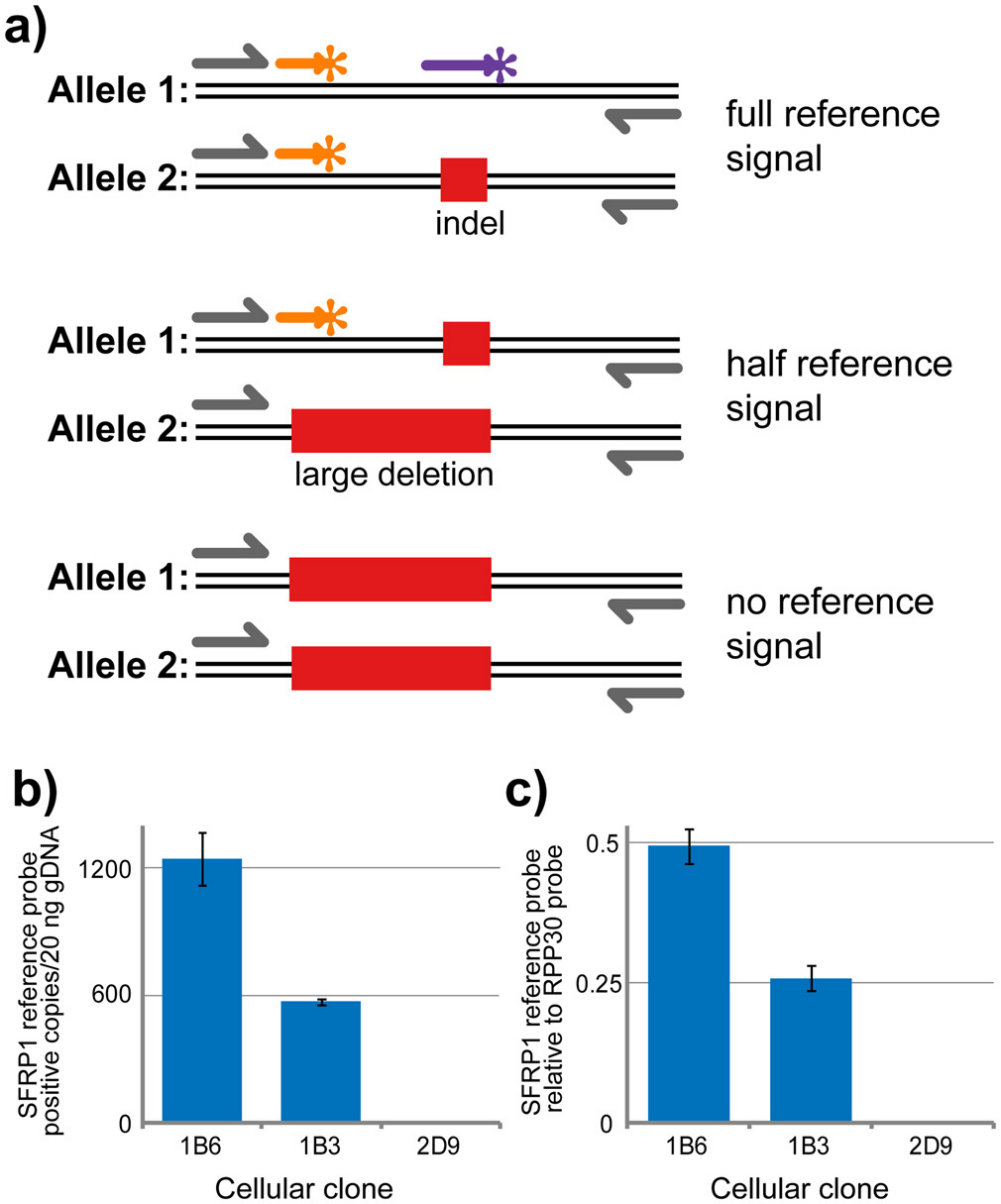
Genomic copy number alterations can be detected in ddPCR NHEJ detection assay. (a) Conceptual diagram displaying the expected changes in reference signal strength dependent on large deletions encompassing the reference probe binding site. (b) SFRP1 reference signal strength (copies/uL) in three C8161 *SFRP1*-edited cellular clones. (c) Copy number analysis of *SFRP1* in three *SFRP1*-edited cellular clones normalized against *RPP30*. Error bars represent standard deviation (n = 3).

The quantitative power of the ddPCR assays led us to test their potential utility in other ways. First, we were interested if they were also capable of identifying large deletions that are occasionally obtained in genome editing experiments. If a deletion extends into any of the reference probe, forward primer, or reverse primer binding sites, these alleles should yield droplets that are also negative for the reference probe in addition to the NHEJ/drop-off probe. Indeed, when we tested gDNA from single cell-derived clones with either one or both *SFRP1* alleles containing a large deletion, the number of total *SFRP1* copies detected was 50% of the wild type for the single large deletion, and 0% (no copies detected) of the wild type for the sample with two large deletion-containing alleles (Fig 4).

**Fig 5:**
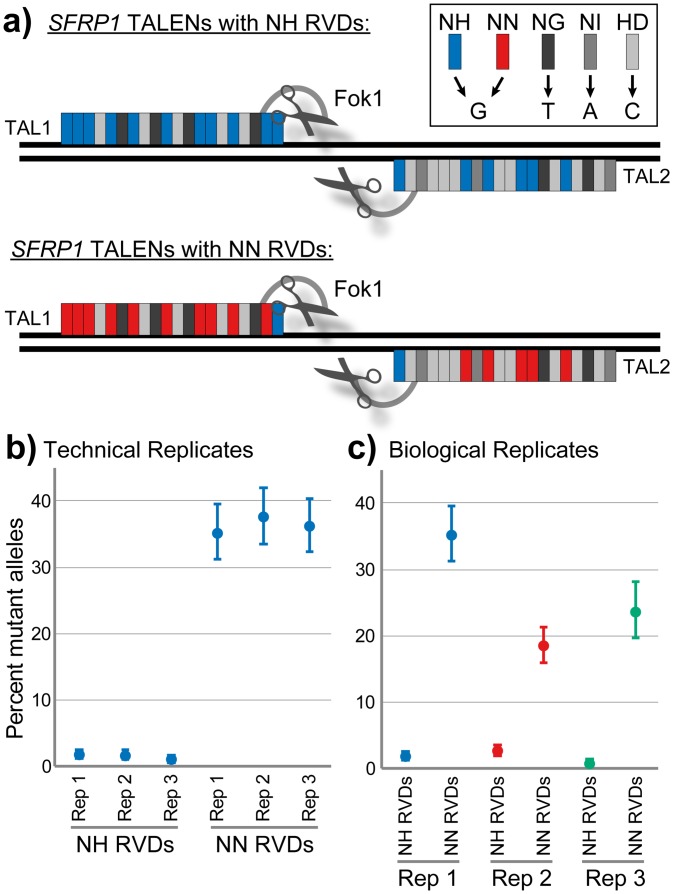
Application of ddPCR NHEJ detection assay to determine genome editing efficiencies of NH versus NN RVD-containing TALENs. (a) TALENs were designed against an *SFRP1* target; NH and NN RVDs were used as illustrated. (b,c) Mutant alleles were detected by the ddPCR NHEJ detection assay in bulk population gDNA of C8161 *SFRP1*-edited cells. Technical (b) and biological (c) replicates illustrate the enhanced gene editing ability of the NN RVD-containing TALEN pair to this target. Points represent percent mutant alleles detected; error bars represent 95% confidence intervals.

Second, we demonstrated that these assays can be used as a quantitative platform to directly test the performance of distinct genome editing systems. We chose to compare the efficiency of TALEN-induced mutations using either NN or NH repeat variable diresidues (RVDs) to bind G nucleotides at the *SFRP1* locus. NN was initially the preferred RVD for the targeting of guanine (G) nucleotides since it was recognized as a “strong repeat” important for the efficacy of TALEN constructs [18]. However, NN was also known to lack desired single nucleotide specificity as it also efficiently recognized adenine (A) nucleotides [19]. More recently, the NH RVD was adopted as an NN substitute to achieve increased specificity for guanine targeting [18,20]. Using the first coding exon of *SFRP1* as an example target locus, our ddPCR assay was used to demonstrate that the NN-containing TALEN pair was much more efficient at inducing mutations than its NH-containing counterpart across three independent experiments (P = 0.0084 by t-test) (Fig 5). An average of 26% of alleles were mutated using the NN-containing TALEN pair, compared to an average of 2% of alleles when the NH-containing TALEN pair was employed. We demonstrate that the technical variability in this assay is minimal, and has the benefit of a statistical confidence interval associated with each individual well, making it possible to quantify potentially small differences in mutation frequency between samples.

## Discussion

Efficient and quantitative screening of nuclease-edited cells is imperative for genome editing to reach its full research potential. We have demonstrated the utility of droplet digital PCR assays for the extremely sensitive detection of indel mutations at target sites using genomic DNA samples from genome editing experiments across different targets and cell lines. We have shown that these assays are specific, quantitative, sensitive, and can serve as a quantitative platform for optimization of genome editing protocols. In addition to their improved accuracy, ddPCR assays offer practical advantages over traditionally employed mismatch nuclease assays. First, only a small amount of gDNA (as little as 5 ng total gDNA) is required for analysis. Second, these assays easily discriminate between single-cell derived clones with a single mutated allele and those with both alleles successfully mutated by NHEJ-derived indels. These characteristics translate to a much more rapid and efficient workflow for the user, and the ability to more quickly focus on clones with complete disruption of gene function.

We presented data for both an ideal assay in *SFRP1* and more challenging assays for two adjacent *NODAL* targets that can be amplified using the same forward and reverse primers. The *SFRP1* assay consistently demonstrated excellent separation between wild type and mutant allele droplet clusters and showed close clustering of droplets in each population (Fig 2A). We never observed any droplets from wild type samples clustering with droplets from double mutant samples and vice-versa. We also observed intermediate droplets that likely represent droplets containing both mutant and wild type DNA molecules at a very low frequency. Even if intermediate droplets are present at a higher frequency, the impact of the user’s threshold choice on quantification is effectively negligible, as is generally true for ddPCR assays.

The *NODAL* assays required some optimization (see methods). Despite being presented with a challenging target, the ddCPR assays still performed extremely well as precise, sensitive, and quantitative screening assays (Fig 3). For *NODAL*, a longer “3-step” thermal cycling protocol was used (see methods). This protocol greatly improved the separation of positive droplets from negative droplets in both the FAM and HEX channels. It is possible that the *NODAL* first exon locus required this optimization due to a very high GC content of 69%. Although distinct, the droplet clusters for wild type and mutant alleles showed less separation and more dispersed clustering within each population. We occasionally observed droplets from wild type samples clustering with droplets from mutant samples, although these were extremely low in frequency and had virtually no impact on mutation quantification within typical experimental ranges. The *NODAL* assays also demonstrated that the HEX and FAM fluorophores are interchangeable in terms of their use as the reference or NHEJ probes. This is useful given that many users may already have a probe-based assay for their target locus and can easily incorporate a second probe of either fluorophore into a duplexed assay for the purpose of mutation screening.

Since ddPCR assays do not require large amplicons, we were able to design our *NODAL* ddPCR assays so as to avoid a nearby heterozygous SNP (S1 Fig). In a mismatch assay for the same locus, unedited wild type *NODAL* amplicons still yielded undesired false positive digestion products. This occurred using both T7E1 and Surveyor nucleases, although T7E1 demonstrated fewer cleavage products. T7E1 nuclease is known to be less sensitive to single base mismatch detection and more sensitive to mismatches resulting from indel mutations [13]. These differences together with a superior signal-to-noise ratio [13], led us to perform our mismatch cleavage assays with T7E1. Even if a heterozygous SNP is contained within a ddPCR assay amplicon, as long as it is not within any of the primer or probe binding sites, it will not contribute to a mutant signal in wild type samples as it does for the mismatch nuclease assays. In this fashion, a potential heterozygous SNP locus can be more easily avoided as the location of probe binding sites is flexible and their footprints are relatively small. For mismatch nuclease assay design, it is often not possible to pick a target for genome editing that is not within several hundred base pairs of a potential heterozygous SNP locus or endogenous mutation—especially in genetically unstable cancer cell lines. Taken together, these aspects of the *NODAL* assay illustrate the versatility of ddPCR mutation screening assays in their ability to be adapted to different target loci of interest.

Perhaps the most appealing aspect of ddPCR mutation screening assays is their sensitivity. This has important implications for experimental workflow. We found that reliable mutation detection could be obtained with a very low amount of input gDNA (e.g. 5 ng or less) This allows samples obtained from a few thousand cells to be analyzed without prior quantification of the gDNA. This is beneficial when screening many single cell-derived clones, as a sample obtained from a single well of a 96 well plate is sufficient for analysis. In contrast, a relatively large amount of input DNA (e.g. 500 ng) is generally required to generate 400 ng of purified target PCR product. Not needing to scale up numerous clones to larger culture vessels is a highly appealing aspect of any genome editing workflow. In addition, despite the requirement for more abundant starting material, we and others [13] have demonstrated that mutation rates as high as 5% are often difficult to detect accurately using mismatch nuclease assays.

Importantly, all of the samples used in this study were genomic DNA preparations and not highly purified PCR products, synthetic oligos, or gene fragments. This allowed us to test the practical utility of these assays in prototypical samples. The theoretical limit of sensitivity for any mutation detection assay is detection of a single mutated molecule in a high background of wild type molecules. In two of our three targets tested, our assays were able to distinguish only 20 pg of mutant DNA from 100 ng of wild type DNA (0.02%). Given that a typical diploid human cell is estimated to contain about 6 pg of gDNA and we were using karyotypically abnormal cancer cell lines, it is likely that 20 pg is very close to the biological limit of detection, representing all of the alleles from a single cell. Remarkably, two of our three assays detected 1.6 and 4.6 copies of mutant gDNA when 20 pg of mutant gDNA was loaded, while only one assay failed to detect mutant gDNA in a 20 pg sample. Due to sampling error and the Poisson statistic used to calculate the number of copies detected in ddPCR assays, if more precision in the quantification of extremely rare mutations is desired, one can perform several technical replicates of the same sample for pooled analysis.

One potential limitation of ddPCR probe-based assays is false-negatives. There are several scenarios in which probe binding may theoretically not be sensitive to target mutation. First, as in any PCR-based assays, the assay may be unable to detect a large deletion that “wipes out” the target completely. While this is generally a disadvantage, we have shown that ddPCR assays may be used to quantitatively assess the loss of target alleles in single cell derived clones (Fig 4). Second, a single base pair indel within the probe binding site may still permit probe binding. PCR probes are routinely used to discriminate between small sequence differences as subtle as single base pair substitutions, and are likely sensitive to the vast majority of single base pair indel mutations. However, since the full range of target mutations in a genome-editing experiment cannot be known *a priori*, it is possible that in some sequence contexts, single base pair indels will still permit probe binding and thus wild type-like fluorescent signal. To reduce this possibility, ddPCR drop-off/ NHEJ probes were designed with relatively low melting temperatures (56–57°C) relative to typical PCR probes such as the reference probe (60°C) so that even a single base pair indel substantially destabilizes probe-target binding. Even if minimally destabilizing mutations still allow some probe binding, they would likely result in lower fluorescence amplitude, manifesting as droplets distinguishable from those containing wild type target. Third, a desired mutation may theoretically occur within the target gene of interest but outside of the drop-off/ NHEJ probe binding site. Surveys of NHEJ mutation signatures for TALEN and CRISPR/Cas genome editing platforms suggest that mutations entirely outside of the TALEN pair spacer region [5], or not affecting bases within three base pairs of the CRISPR/Cas9 cut site [21] are likely very rare. Therefore, for maximum sensitivity to all possible nuclease-induced mutations, we recommend designing TALEN pairs such that the spacer region is not substantially longer than typical ddPCR drop-off/ NHEJ probes (approximately 15–20 base pairs). For CRISPR/Cas9 assays, we recommend a drop-off/ NHEJ probe binding site that is centered on (or very close to) the predicted cut site (three bases upstream/ 5’ of the Cas9 ‘NGG’ protospacer adjacent motif or ‘PAM’). These recommendations allow sensitivity to the vast majority of nuclease-induced mutations. Using these guidelines, we demonstrated that our “Nodal target 2” assay was fully sensitive to a single base-pair deletion mutation (Figs 3B and 2C), and was able to accurately quantify this mutant in a high background of wild type DNA (Fig 3E). We did not encounter any sequence mutations in samples identified as wild type by our ddPCR assays. In summary, ddPCR is an extremely sensitive mutation detection method.

We also used this assay to demonstrate improved genome editing performance of TALENs containing the NN RVD relative to those consisting of NH to target guanine for an *SFRP1* target. In addition to highlighting the ability of ddPCR assays to serve as a quantitative screening platform, these data also suggest that it may not be advisable to use the NH RVD in some target contexts. Indeed, widely followed design guidelines [18] available as options in TALEN design software and assembly kits [22,23] suggest to target loci with at least 25% C+G and avoid stretches of 6 or more A+T. This recommendation was initially made based on the identification of NN (targeting G) and HD (targeting C) as “strong binders” that stabilized TALEN-DNA binding [18]. However, since these recommendations were published, NH has become widely adopted as the G-targeting RVD of choice due to increased specificity over NN [18,20,24]. Unfortunately, the strength of NH binding appears to be context dependent and has been characterized as an “intermediate binder.” Specifically, unlike NN, using NH to target G did not result in any TALEN activity for an A+ T rich 11 bp target lacking any C nucleotides [18]. If the design guidelines of >25% C+G are extended to “maximize C+G” and the NH RVD is employed, we hypothesized that TALEN activity may suffer. Indeed, this was the case for our *SFRP1* target, which is very G rich (Left TALEN target: 11/20 = 55% G). Therefore we suggest that great thought be given to which approach is appropriate for the targeting of G nucleotides at a given target.

## Conclusions

We have demonstrated that droplet digital PCR mutation detection assays have great utility and offer several benefits over conventional mutation screening methods. They are ideal for rapid genome editing workflows as they require very little sample genomic DNA, and the same assay can be used for screening bulk populations and single cell-derived clones. These assays will undoubtedly continue to increase in popularity and contribute to rapid and quantitative genome editing workflows.

## Methods

### Cell culture

MCF7 human breast cancer cells (ATCC) and C8161 human melanoma cells [25] were used for *NODAL* targeting and *SFRP1* targeting experiments, respectively. Cells were cultured in RPMI supplemented with 10% FBS (Thermo Fisher) and incubated at 37°C and 5% CO_2_ in humidified chambers.

### Plasmid generation

“All-in-one CRISPR/Cas9 LacZ” was generated from the “scrambled sgRNA control for pCRISPR-CG01” plasmid (Genecopedia). Two unique Esp3I restriction sites flanking the gRNA sequence were consecutively introduced using the QuikChange Lightning Site-Directed Mutagenesis Kit (Agilent). The LacZα fragment was then cloned into the plasmid using BsmBI (an isoschizomer of Esp3I) (NEB), replacing the original gRNA. The All-in-one CRISPR/Cas9 LacZ plasmid and complete sequence information is available from Addgene. This plasmid is ready for one-step cloning (protocol in S1 File).

### TALEN design and cloning

TALEN targets were designed using the TAL Effector Nucleotide Targeter 2.0 (https://tale-nt.cac.cornell.edu/node/add/talen) [22,23], using either NH or NN to target G nucleotides, the Streubel et al. guidelines “on,” and the upstream base as “T only.” The *SFRP1* target sequences can be found in supporting figure 1 (S1 Fig).

TALENs were assembled using the Golden Gate TALEN and TAL Effector Kit 2.0, Addgene kit # 1000000024 (https://www.addgene.org/taleffector/goldengatev2/) using either the NN or NH RVD to target G nucleotides. In cases where the most 3’ nucleotide was G, NH (and not NN) was always used as the last half repeat. We used pTAL7a (Addgene plasmid #48705) for the “left”/ sense-targeting TALEN, and pTAL7b (Addgene plasmid #48706) for the “right”/ antisense-targeting TALEN as final destination plasmids [26].

### TALEN transfection and single cell clone generation

C8161 cells were transfected with TALEN pairs using GeneIn transfection reagent (Global Stem). For a single well of a 6 well dish, 3 ug plasmid DNA (1.5 ug TAL1-containing plasmid, 1.5 ug TAL2-containing plasmid), 8 uL of red reagent, 8 uL of blue reagent, and 400 uL of OptiMEM was used. To enrich for edited cells, 48 hours post-transfection, cultures were dual selected with puromycin (0.5 ug/mL) and Blasticidin 2 ug/mL) for six days. Cultures were expanded for seven days before genomic DNA extraction. To obtain single cell clones, a 96 well plate was plated with a filtered cell suspension (40 uM filter, Thermo Fisher) at a concentration of 0.5 cells/well.

### CRISPR transfection and single cell clone generation

CRISPR-Cas plasmids used for transfection were either purchased as custom gRNAs in the CG01 plasmid backbone (Genecopedia) or generated from the modified “All-in-one CRISPR/Cas9 LacZ” plasmid. The *NODAL* target sequences can be found in supporting figure 1 (S1 Fig). CRISPR-Cas plasmids were transfected using GeneIn transfection reagent (Global Stem). For a single well of a 12 well dish, 2 ug plasmid DNA, 8uL of red reagent, 4 uL of blue reagent, and 200 uL of OptiMEM was used. To enrich for edited cells, cultures were either sorted for mCherry+ cells using flow cytometry (Faculty of Medicine and Dentistry Flow Cytometry Facility at the University of Alberta), or selected for 10–14 days using 600–1000 ug/mL Geneticin (Thermo Fisher). Single-cell derived clones were generated using either flow cytometry to plate a single cell per well of a 96 well plate, or filtered using a 40 uM filter (Thermo Fisher) and plated at a concentration of 0.5 cells/ well.

### Genomic DNA isolation

Genomic DNA was isolated using the PureLink Genomic DNA isolation kit (Thermo Fisher) and quantified using the Epoch Microplate Spectrophotometer (BioTek).

### Droplet digital PCR

Droplet digital PCR assays consisted of the following components (final concentrations in 20 uL total reaction volume): ddPCR SuperMix for Probes (no dUTP) (1x, Bio-Rad), forward primer (900 nM), reverse primer (900 nM), Reference probe (FAM or HEX, 250 nM), NHEJ/drop-off probe (different fluorophore than reference; FAM or HEX, 250 nM), restriction enzyme (AluI, 4 units), nuclease-free water, and gDNA. All primers and probes were designed using Primer3 plus (http://primer3plus.com) and purchased from IDT DNA. All probes included the ZEN internal quencher and 3’ Iowa Black FQ quencher. All ddPCR assays were analyzed using the QX200 droplet reader and Quantasoft software version 1.7.4 (Bio-Rad). Standard ddPCR thermal cycling conditions were used for the *SFRP1* assay, with an annealing temperature of 55°C. For *NODAL* assays, a “3-step” protocol was used, with an annealing temperature of 56°C and an additional 2 minute extension step at 72°C performed for each cycle. For copy number analysis, an RPP30 assay (Bio-Rad; dHsaCP1000485) was used as a reference and *SFRP1* FAM assays were performed in parallel with 20 ng of gDNA loaded for each reaction.

### ddPCR assay primer and probe sequences

NODAL mismatch forward primer: TCCCCAGAGGGAGGAAAGG

NODAL mismatch reverse primer: CAGGCTCCGGGATAAGCAAC

NODAL ddPCR forward primer: TTCCTTCTGCACGCC

NODAL ddPCR reference probe:

TGGGCCCTACTCCAGG (/5HEX/TGG GCC CTA /ZEN/CTC CAG G/3IABkFQ/)

NODAL ddPCR target 1 drop-off/ NHEJ probe:

CCGCGTACGCAGGAGC (/56-FAM/CCG CGT ACG /ZEN/CAG GAG C/3IABkFQ/)

NODAL ddPCR target 2 drop-off/ NHEJ probe:

CTCAGCATGTACGCCAGAG (/56-FAM/CTC AGC ATG /ZEN/TAC GCC AGA G/3IABkFQ/)

NODAL ddPCR reverse primer: TAGGCTGCGGATGATG

SFRP1 mismatch forward primer: CGAGGGCCGCCACTG

SFRP1 mismatch reverse primer: TTCACCTCCGCCATGGTCTC

SFRP1 ddPCR forward primer: CATGGGCATCGGGCG

SFRP1 ddPCR reference probe:

CTGGGCGTGCTGCTGG (/56-FAM/CTG GGC GTG /ZEN/CTG CTG G/3IABkFQ/)

SFRP1 ddPCR drop-off/ NHEJ probe:

CGCGGCGCTTCTGGC (/5HEX/CGC GGC GCT /ZEN/TCT GGC /3IABkFQ/)

SFRP1 ddPCR reverse primer: CGTAGTCGTACTCGCTGG

The binding sites for primers and probes for both loci are illustrated in supporting figure 1 (S1 Fig).

### ddPCR dilution series

For the ddPCR dilution series in Fig 3, negative control (wild-type only) wells and positive control (mutant only) wells were used to assign thresholds for all dilution sample wells. The wild type population was quantified by setting all other droplets as FAM-negative and HEX-negative. The NHEJ population was quantified manually using the equation: copies/ 20 uL sample = -ln(1-p) x 20,000 / 0.85. ‘p’ is the proportion of positive droplets defined as NHEJ droplets/ (NHEJ droplets + empty droplets), and 0.85 nL is the average volume of a droplet as used by QuantaSoft (Bio-rad) [27]. Note that for quantification of NHEJ, wild type droplets are excluded from the calculation, as an indistinguishable subpopulation of wild type droplets will also contain NHEJ targets. Future versions of QuantaSoft (Bio-rad) will allow for more automated quantification of multiple droplet populations within the same sample. The expected number of copies was calculated based on the number of copies detected by ddPCR in 100 ng (as measured by spectrophotometry) of each mutant sample. Double amounts of mutant gDNA were loaded for the SFRP1 dilution series, since one copy of the SFRP1 target region was not detected in this sample (Fig 4).

### T7E1 assays

For T7E1 mismatch assay, genomic DNA was isolated and PCR amplified using AmpliTaq Gold 360 Master Mix (Thermo Fisher). PCR products were purified using the PureLink PCR Purification Kit (Thermo Fisher) and quantified using the Epoch Microplate Spectrophotometer (BioTek). 400 ng of purified PCR product was used in an annealing reaction and T7E1 digestion (New England BioLabs) as previously described [28]. Cleavage was visualized by agarose gel electrophoresis and detection using the AlphaImager HP (Bio-techne). Band intensities were obtained by AlphaView software (Bio-techne). Analysis bands were placed so as to completely encompass each visible band. Where bands were difficult to visualize, analysis bands were placed in the same location as adjacent wells to provide an unbiased quantification. All analysis bands for bands of a given size were the same width across all lanes. The detected percent digested was calculated as the sum of the intensities of the digested fragment bands divided by the sum of the intensities of all bands. The expected percent digested was determined by assuming random hybridization of alleles and determining the expected frequency of heteroduplexes. For example, in the 50:50 sample for NODAL target 2, there are three distinct alleles (one wild type and two mutant) at frequencies of 50%, 25%, and 25%. Unrecognized homoduplexes are expected at rates of 0.5 x 0.5 = 0.25, 0.25 x 0.25 = 0.0625 and 0.25 x 0.25 = 0.0625, for a total of 37.5%. Thus the remaining heteroduplexes are expected to constitute 62.5% of the total.

### Sequencing of single-cell clones

For sequencing of single cell clones, gDNA was isolated and PCR amplified using AmpliTaq Gold 360 Master Mix (Thermo Fisher) and the forward and reverse primers from either the ddPCR or mismatch nuclease assay. PCR products were cloned using the TOPO TA Cloning Kit (Thermo Fisher), minipreped using the Diamed High-Speed Plasmid Mini Prep Kit (Frogga Bio), and Sanger sequenced using the M13R primer that binds the pCR-4-TOPO backbone.

### ddPCR NHEJ mutation screening assay design guidelines

ddPCR assays were designed using Primer3Plus (http://primer3plus.com) with modified settings: 50 mM monovalent cations, 3.0 mM divalent cations, 0 mM dNTPs, and SantaLucia 1998 thermodynamic and salt correction parameters. Predicted nuclease cut sites were positioned mid-amplicon, with 75–125 bp flanking either side up to the primer binding sites. Reference probe and primers were designed distant from the cut site (origin of NHEJ generation). Optimal annealing temperatures were determined empirically by temperature gradient. In general, we recommend designing primers with T_m_ = 55°C, reference probes with T_m_ = 60°C, and NHEJ/drop-off probes with T_m_ = 56–57°C. However, higher melting temperatures are appropriate for high-GC targets to design primers and probes of sufficient length.

## Supporting Information

S1 File Instructions for one-step gRNA cloning into All-in-one CRISPR/Cas9 LacZ. (DOCX)

S1 Fig Sequence maps of (a) *NODAL* and (b) *SFRP1* loci depicting locations of primers, probes, and additional features as indicated for mismatch and ddPCR assays. (TIF)

## Abbreviations

Cas: CRISPR-associated, genes
CRISPR: clustered regularly interspaced short palindromic repeats
ddPCR: droplet digital polymerase chain reaction
gDNA: genomic deoxyribonucleic acid
gRNA: guide ribonucleic acid
HDR: homology-directed repair
NHEJ: non-homologous end joining
PAM: Protospacer adjacent motif
RVD: repeat variable diresidue
TALEN: Transcription activator-like effector nuclease

## References

[1] Joung JK, Sander JD. TALENs: a widely applicable technology for targeted genome editing. Nat Rev Mol Cell Biol. 2012;14: 49–55. 10.1038/nrm3486 23169466 PMC3547402

[2] Sun N, Zhao H. Transcription activator-like effector nucleases (TALENs): A highly efficient and versatile tool for genome editing. Biotechnol Bioeng. 2013 10.1002/bit.24890 23508559

[3] Ran FA, Hsu PD, Wright J, Agarwala V, Scott DA, Zhang F. Genome engineering using the CRISPR-Cas9 system. Nat Protoc. 2013;8: 2281–2308. 10.1038/nprot.2013.143 24157548 PMC3969860

[4] Doudna JA, Charpentier E. The new frontier of genome engineering with CRISPR-Cas9. Science. 2014;346: 1258096–1258096. 10.1126/science.1258096 25430774

[5] Kim Y, Kweon J, Kim J-S. TALENs and ZFNs are associated with different mutation signatures. Nat Meth. 2013;10: 185 10.1038/nmeth.2364 23396284

[6] Sung YH, Kim JM, Kim HT, Lee J, Jeon J, Jin Y, et al Highly efficient gene knockout in mice and zebrafish with RNA-guided endonucleases. Genome Res. 2014;24: 125–131. 10.1101/gr.163394.113 24253447 PMC3875853

[7] Flemr M, Bühler M. Single-Step Generation of Conditional Knockout Mouse Embryonic Stem Cells. Cell Reports. 2015;12: 709–716. 10.1016/j.celrep.2015.06.051 26190102

[8] Shalem O, Sanjana NE, Hartenian E, Shi X, Scott DA, Mikkelsen TS, et al Genome-scale CRISPR-Cas9 knockout screening in human cells. Science. American Association for the Advancement of Science; 2014;343: 84–87. 10.1126/science.1247005 PMC4089965 24336571

[9] Yang Z, Steentoft C, Hauge C, Hansen L, Thomsen AL, Niola F, et al Fast and sensitive detection of indels induced by precise gene targeting. Nucleic Acids Research. 2015;43: e59–e59. 10.1093/nar/gkv126 25753669 PMC4482057

[10] Yu C, Zhang Y, Yao S, Wei Y. A PCR Based Protocol for Detecting Indel Mutations Induced by TALENs and CRISPR/Cas9 in Zebrafish. Jennings B, editor. PLoS ONE. 2014;9: e98282–7. 10.1371/journal.pone.0098282 24901507 PMC4046980

[11] Hendel A, Fine EJ, Bao G, Porteus MH. Quantifying on- and off-target genome editing. Trends Biotechnol. 2015;33: 132–140. 10.1016/j.tibtech.2014.12.001 25595557 PMC4308725

[12] Wang K, Mei DY, Liu QN, Qiao XH, Ruan WM, Huang T, et al Research of methods to detect genomic mutations induced by CRISPR/Cas systems. J Biotechnol. 2015;214: 128–132. 10.1016/j.jbiotec.2015.09.029 26419205

[13] Vouillot L, Thélie A, Pollet N. Comparison of T7E1 and surveyor mismatch cleavage assays to detect mutations triggered by engineered nucleases. G3 (Bethesda). 2015;5: 407–415. 10.1534/g3.114.015834 25566793 PMC4349094

[14] Mock U, Machowicz R, Hauber I, Horn S, Abramowski P, Berdien B, et al mRNA transfection of a novel TAL effector nuclease (TALEN) facilitates efficient knockout of HIV co-receptor CCR5. Nucleic Acids Research. 2015;43: 5560–5571. 10.1093/nar/gkv469 25964300 PMC4477672

[15] Miyaoka Y, Chan AH, Judge LM, Yoo J, Huang M, Nguyen TD, et al Isolation of single-base genome-edited human iPS cells without antibiotic selection. Nat Meth. 2014;11: 291–293. 10.1038/nmeth.2840 PMC4063274 24509632

[16] Quail DF, Siegers GM, Jewer M, Postovit L-M. Nodal signalling in embryogenesis and tumourigenesis. The International Journal of Biochemistry & Cell Biology. 2013 10.1016/j.biocel.2012.12.021 23291354

[17] Surana R, Sikka S, Cai W, Shin EM, Warrier SR, Tan HJG, et al Secreted frizzled related proteins: Implications in cancers. Biochim Biophys Acta. 2014;1845: 53–65. 10.1016/j.bbcan.2013.11.004 24316024

[18] Streubel J, Blücher C, Landgraf A, Boch J. TAL effector RVD specificities and efficiencies. Nature Biotechnology. 2012;30: 593–595. 10.1038/nbt.2304 22781676

[19] Boch J, Scholze H, Schornack S, Landgraf A, Hahn S, Kay S, et al Breaking the code of DNA binding specificity of TAL-type III effectors. Science. 2009;326: 1509–1512. 10.1126/science.1178811 19933107

[20] Cong L, Zhou R, Kuo Y-C, Cunniff M, Zhang F. Comprehensive interrogation of natural TALE DNA-binding modules and transcriptional repressor domains. Nat Comms. 2012;3: 968 10.1038/ncomms1962 PMC3556390 22828628

[21] Bell CC, Magor GW, Gillinder KR, Perkins AC. A high-throughput screening strategy for detecting CRISPR-Cas9 induced mutations using next-generation sequencing. BMC Genomics. 2014;15: 1002 10.1186/1471-2164-15-1002 25409780 PMC4246457

[22] Doyle EL, Booher NJ, Standage DS, Voytas DF, Brendel VP, VanDyk JK, et al TAL Effector-Nucleotide Targeter (TALE-NT) 2.0: tools for TAL effector design and target prediction. Nucleic Acids Research. 2012;40: W117–W122. 10.1093/nar/gks608 22693217 PMC3394250

[23] Cermak T, Doyle EL, Christian M, Wang L, Zhang Y, Schmidt C, et al Efficient design and assembly of custom TALEN and other TAL effector-based constructs for DNA targeting. Nucleic Acids Research. 2011;39: e82–e82. 10.1093/nar/gkr218 21493687 PMC3130291

[24] Jankele R, Svoboda P. TAL effectors: tools for DNA Targeting. Briefings in Functional Genomics. 2014;13: 409–419. 10.1093/bfgp/elu013 24907364 PMC4168661

[25] Welch DR, Bisi JE, Miller BE, Conaway D, Seftor EA, Yohem KH, et al Characterization of a highly invasive and spontaneously metastatic human malignant melanoma cell line. Int J Cancer. 1991;47: 227–237. 1671030 10.1002/ijc.2910470211

[26] Frank S, Skryabin BV, Greber B. A modified TALEN-based system for robust generation of knock-out human pluripotent stem cell lines and disease models. BMC Genomics. 2013;14: 773 10.1186/1471-2164-14-773 24206569 PMC3840567

[27] Corbisier P, Pinheiro L, Mazoua S, Kortekaas A-M, Chung PYJ, Gerganova T, et al DNA copy number concentration measured by digital and droplet digital quantitative PCR using certified reference materials. Anal Bioanal Chem. 2015;407: 1831–1840. 10.1007/s00216-015-8458-z 25600685 PMC4336415

[28] Lin Y, Cradick TJ, Bao G. Designing and Testing the Activities of TAL Effector Nucleases In: Storici F, editor. Gene Correction. Totowa, NJ: Humana Press; 2014 pp. 203–219. 10.1007/978-1-62703-761-7_13 24557905

