# Supplementary material for: A Digital PCR-Based Method for Efficient and Highly Specific Screening of Genome Edited Cells
Source: PLoS One. 2016 Apr 18;11(4):e0153901. doi: 10.1371/journal.pone.0153901 (PMC4835065; doi:10.1371/journal.pone.0153901)
Supplement: S1 File — (DOCX) [file pone.0153901.s001.docx]

**Instructions for one-step gRNA cloning into All-in-one CRISPR/Cas9 LacZ**

1. Design an appropriate gRNA for your target of interest using software of choice.
2. Order a g-Block (IDT DNA) that includes your gRNA sequence. Replace all of the 20 “N” bases an 18-20 bp gRNA sequence in the 5’-3’ orientation (as supplied by the design tools). Include a 5’ G if desired. Do not leave any “N” bases in the sequence. Double check that this new sequence does not introduce a new Esp3I restriction site (“CGTCTC” or “GAGACG”). This is unlikely, but would interfere with cloning. There should be only two such sites in the whole g-Block sequence.

>example_g_block_insert_for_crispr_all_in_one

ATATATCGTCTCGAACTTGAAAGTATTTCGATTTCTTGGGTTTATATATCTTGTGGAAAGGACGAAACACCNNNNNNNNNNNNNNNNNNNNGTTTTAGAGCTAGAAATAGCAAGTTAAAATAAGGCTAGTCCGTTATCAACTTGAAAAAGTGGCACCGAGTCGGTGCTTTTTTCTAGACACAATTGCATGAAGAATCTGCTTAGGGTTAGGCGTTTTGCGCTAGAGACGAATTAT

1. Re-suspend g-Block in TE buffer to a final concentration of 10 ng/uL.
2. Mix the following components in a 0.2 mL PCR tube:
   1. g-Block 25 ng
   2. All-in-one CRISPR/Cas9 LacZ 75 ng
   3. BsmBI (10 U/uL) 1 uL
   4. T4 ligase (Thermo Fisher: 15224017) 1 uL
   5. T4 buffer 2 uL
   6. Nuclease-free water to 20 uL total

5) Incubate in a standard thermal-cycler using the following conditions:

- 1. 37° 5 min
  2. 16° 10 min
  3. 37° 15 min
  4. 80° 5 min

The reaction is now ready for transformation (use a maximum of 5 uL for 50 uL competent cells) and plasmid preparation. Colonies containing successfully cloned plasmids will be white if using blue/white screening. Selected clones can be sequenced using the SP6F primer.
