# Supplementary figures and images for: A Digital PCR-Based Method for Efficient and Highly Specific Screening of Genome Edited Cells
Source: PLoS One. 2016 Apr 18;11(4):e0153901. doi: 10.1371/journal.pone.0153901 (PMC4835065; doi:10.1371/journal.pone.0153901)

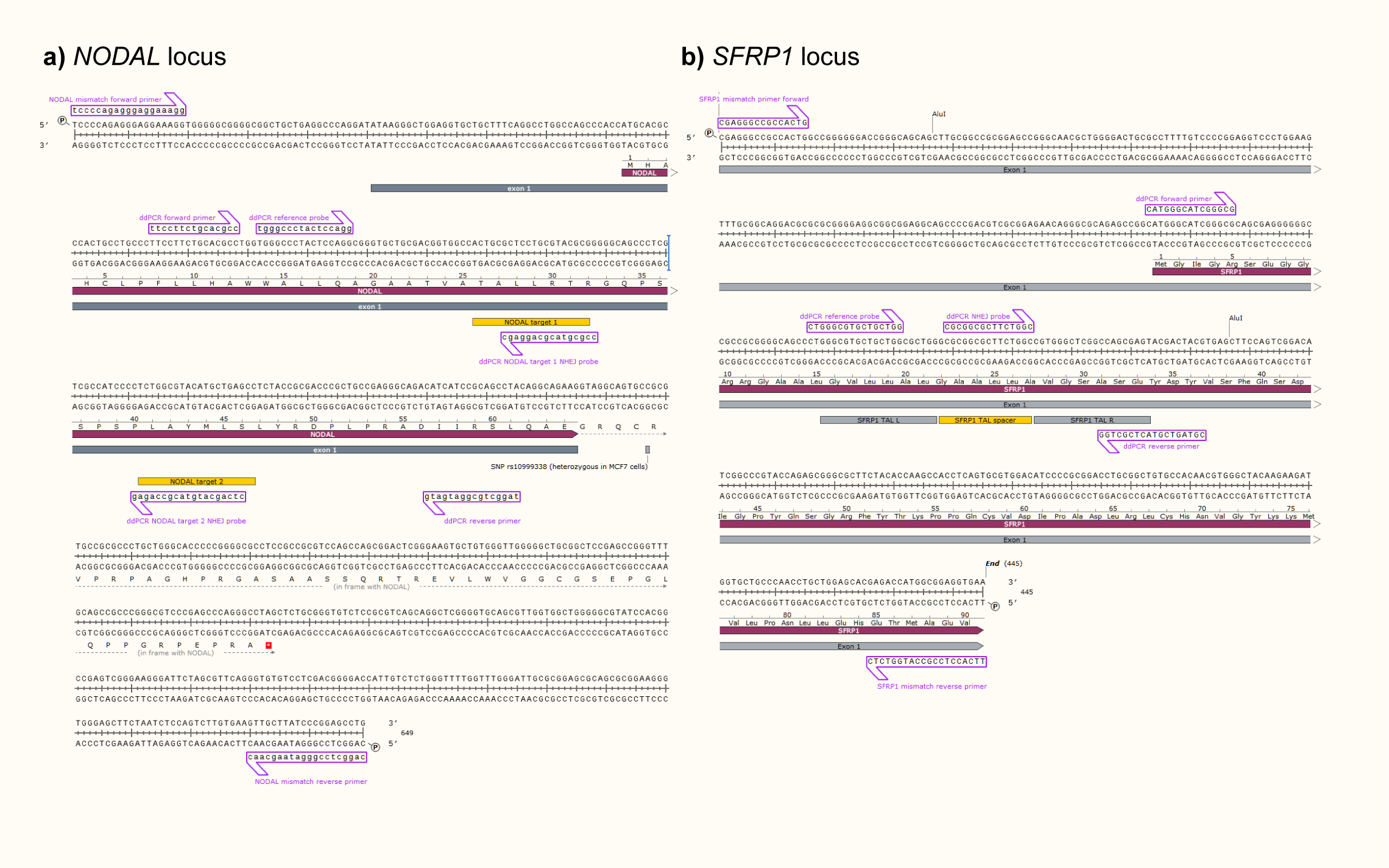

Supplement: S1 Fig — (TIF) [file pone.0153901.s002.tif]
